# Seasonal modifications of longitudinal distribution patterns within a stream: Interspecific interactions in the niche overlap zones of two *Ephemera* mayflies

**DOI:** 10.1002/ece3.8766

**Published:** 2022-04-01

**Authors:** Seiya Okamoto, Masaki Takenaka, Koji Tojo

**Affiliations:** ^1^ 222722 Division of Mountain and Environmental Science Interdisciplinary Graduate School of Science and Technology Shinshu University Matsumoto Japan; ^2^ Sugadaira Research Station Mountain Science Center University of Tsukuba Ueda Japan; ^3^ 222722 Department of Biology Faculty of Science Shinshu University Matsumoto Japan; ^4^ 222722 Institute of Mountain Science Shinshu University Matsumoto Japan

**Keywords:** Ephemeroptera, habitat segregation, Interspecific competition, niche differentiation, river system

## Abstract

Niche differentiation between closely related species leads to differentiation of their habitats. Segregation based on slight differences in environmental factors, that is niche differentiation on the microhabitat scale, allows more species to inhabit a certain geographic space. Therefore, such fine scale niche differentiation is an important factor in the support of species diversity. In addition, niche differentiation on the microhabitat scale and/or the differentiation of breeding seasons can be considered typical mechanisms that facilitate multispecies’ co‐existence. In this study, sister species (Commonly, *Ephemera japonica* inhabit at upstream region and *Ephemera strigata* inhabit at middle stream region), which often coexist in the upper to middle reaches of river systems of the Japanese Islands, were targeted and the following aspects were investigated. First, differences in habitat preference and interspecific differences in flow distribution patterns on a geographically fine scale were tracked in detail. Subsequently, the temporal transitions of their distribution patterns were investigated in detail and seasonal changes were investigated. Finally, we thoroughly investigated the disappearance of nymphs of each species from the river due to emergence affected the distribution of each species (by conducting daily emergence surveys). Combining results of these multiple studies also suggested that there may be spatiotemporal interspecial interaction between these two species within/around their overlapping regions. Traditionally, the longitudinal distribution pattern of these two *Ephemera* mayflies has been thought to be established based on a difference in habitat preferences, but this study revealed that the interspecific interaction between the two species also plays an important role. This study provides new insights into species diversity and distribution pattern formation in river‐dwelling species.

## Graphical abstract

In this study, *Ephemera japonica* and *Ephemera strigata* of sister species, which often coexist in the upper to middle reaches of river systems of the Japanese Islands, were targeted and multiple studies (e.g., seasonal changes of nymphs distribution patterns and surveys of daily adult emergence) were conducted. Combining results of these multiple studies suggested that may be spatiotemporal interspecific interaction between these two species within/around their overlapping regions.

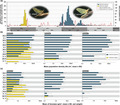

## 1 INTRODUCTION

“Niche differentiation” is one of the important factors that creates biodiversity (Leibold & McPeek, 2006; Takenaka et al., 2021; Tilman, 2000). The finer the niche differentiation that occurs on the microhabitat scale, the greater the degree of biodiversity within the area (Kovalenko et al., 2012; Maltseva et al., 2021). Niche differentiation is closely related to the functional characteristics, the resource used, the habitat preference of a species, and also its relationships with other species (D'Andrea & Ostling, 2016; Michalko & Pekár, 2015; Nicholls & Racey, 2006; Schiesari et al., 2009). Fine‐scale “niche differentiation” between species and intraspecific lineages and the resulting “habitation differentiation” are the main mechanisms of species diversification (Cádiz et al., 2013; Pavlek & Mammola, 2021); these are important subjects in evolutionary ecology. In fact, it is well known that environmental factors are the main factors in establishing longitudinal distribution between interspecific and intraspecific lineages, including among closely related species (e.g., aquatic insects: Hildrew & Edington, 1979; Ogitani et al., 2011; Saito & Tojo, 2016; Saito et al., 2018; freshwater snails: Atkinson et al., 2012; freshwater fish: Morita et al., 2016). Using river systems as a target of study gives an advantage of easy quantitative evaluation of various environmental factors that are closely involved in differentiating the niches of organisms inhabiting rivers such as riverbed slope gradient, flow velocity, water depth, water quality, water temperature, riverbed substrate size, the abundance of organic matter and algae periphyton, and the degree of isolation (Okamoto et al., 2022). Under such circumstances, detailed longitudinal distribution patterns on the microhabitat scale of freshwater animals inhabiting rivers have been frequently discussed (Costa & Melo, 2008; Mauchart et al., 2017; Morita et al., 2016; Ogitani et al., 2011; Okamoto et al., 2022; Roberts & Angermeier, 2007; Saito & Tojo, 2016).

Although abiotic environmental factors have often been targeted as being related to the distribution patterns of freshwater benthic organisms in these discussions (Heino, 2009; Okamoto et al., 2022; Serpa et al., 2020), interspecies interactions have also been emphasized as factors that determine distributions, especially considering important local factors. For example, the following cases are interesting and typical interspecific interactions: the competition for the habitat of a net‐winged midge, *Blepharicera micheneri*, and a black fly, *Simulium virgatum* (Dudley et al., 1990), a filter feeder caddisfly *Glossosoma nigrior* (Kohler, 1997); the predator–prey relationship of a perlid stonefly with a baetid mayfly (Peckarsky et al., 1993); parasitism of nematodes on *Enomus* and other caddisflies (Grabner, 2017; Lancaster & Bovill, 2017); and as a positive interaction between species, the increase in the density of *Ephemerella setigera*, which is caused by a relaxation in the flow velocity by the nesting of a caddisfly, *Hydropsyche orientalis* (Nakano et al., 2005).

In many previous studies of river‐inhabiting species that tend to have a flow‐related distribution tendency, it has been important to understand the relationship between the distribution regions of species/lineages and the major primary related environmental factors (Helson et al., 2006; Illéšová et al., 2008; Lehotský et al., 2016). However, such studies have been conducted on the premise that differences in habitat preference and/or differences in life history between target species are determined according to their distribution ranges. Studies that explore in detail the seasonal changes in distribution ranges between closely related species based on the ecological niche and the role that their interactions play in determining their distributional ranges are rare.

Since it is extremely difficult to evaluate interspecific interactions, it has been the conventional approach to use experimental methods such as confirming the response of the other species when a certain species is artificially excluded (Connell, 1961a, 1961b; Paterson et al., 2018; Robertson, 1996). Such manipulation experiments conducted on stream insects inhabiting running water are basically difficult (however, although it is a special case, there is a research case targeting aquatic insects that adheres to the conditions of a rocky splash zone; Dudley et al., 1990). In this study, we focused on a natural phenomenon in which the nymphs of one of these two closely related species temporarily disappears from their habitat during a certain period, due to the early emergence of one of the species from their cohabitation region. As such, few ecological studies have actually investigated in detail the seasonal changes in aquatic insect distribution patterns, population densities, biomasses, and interactions between closely related species.

As the target group in this study, we focused on an *Ephemera* mayfly group, whose distribution characteristics within the Japanese Archipelago have already been investigated in detail and for which the tendency for fine‐scale niche differentiation within specific river systems has been reported (Okamoto et al., 2022; Okamoto & Tojo, 2021). Regarding the longitudinal distributions of *Ephemera* mayflies, environmental factors have been suggested to have an essential role to play in their distribution and densities, based on the results of our own previous studies (Okamoto et al., 2022; Okamoto & Tojo, 2021). In particular, we focused on *Ephemera japonica* and *Ephemera strigata*, for which there is detailed information on reproductive seasons and emergence patterns. Also, they often overlap in their distribution ranges with each other in the upper reaches of river systems, so it is extremely important to clarify the seasonal changes in the habitat preferences of the target species and the resulting seasonal changes in their distribution areas, taking into account the life history of the targeted species.

Regarding the relative distribution patterns of these two *Ephemera* mayflies, it has already been clarified that *E*. *japonica* inhabits upstream regions and *E*. *strigata* inhabits downstream regions (Okamoto et al., 2022; Okamoto & Tojo, 2021). In addition, it is well known that the distribution areas of these two species widely overlap and their reproductive seasons are staggered (Gose, 1970; Ishiwata & Takemon, 2005; Takemon, 1989, 1990). These two *Ephemera* mayflies are phylogenetically closely related (Tojo & Machida, 1998) and are considered to be sister species with a common ancestry. That is, it is predicted that the current marked longitudinal distribution pattern has evolved as a result of the sister species adapting to different niches.

The purpose of this study was to clarify in detail how environmental factors and interspecific interactions contributed to the longitudinal distribution patterns of these two *Ephemera* mayflies. The following two subjects were investigated in detail by conducting a rigorous spatiotemporal survey to answer two important questions: 1) Is there a seasonal change in the longitudinal distribution patterns of these two species of *Ephemera* mayflies? 2) Do not only environmental factors (abiotic factors) but also interspecific interactions between these two *Ephemera* species influence the determination of their distribution ranges?

## 2 MATERIALS AND METHODS

### 2.1 Targeted species and study sites

The targeted stream, the Metoba‐gawa River, is a small tributary (i.e., stream length approximately 15 km, catchment area approximately 53 km^2^) located in the upper reaches of the Chikuma‐Shinano‐gawa River System, which is one of the largest river systems in Japan. Our preliminary research showed that the two mayfly species, *Ephemera japonica* and *Ephemera strigata* inhabit the Metoba‐gawa River at high densities (as a result of our preliminary specimen sampling using quadrats in March, 2019, the maximum density of *E*. *japonica* was 93 specimens/m^2^; the maximum densities of *E*. *strigata* was 138 specimens/m^2^ with quadrat). Since the Metoba‐gawa River is adjacent to our laboratory, it was possible to conduct daily sample surveys on the level of emergence of the target species over a long period of time (from April to September 2019). In addition, due to the moderate stream size of the Metoba‐gawa River, it is suitable for carrying out quantitative surveys that can cover almost all microhabitats in the stream in order to evaluate the characteristics of these habitats and their relationships to population densities and biomass. In this study, based on the results of a preliminary survey, we selected 15 study sites. These 15 fixed survey points were set to include the upstream zone where only *E*. *japonica* occurred, the downstream zone where only *E*. *strigata* occurred in the preliminary survey, and an intermediate basin where both species coexist within the Metoba‐gawa River. In addition, in order to evaluate the emergence time of both species accurately, the respective research sites were set in locations where the population densities of each species were high during Period A (St. 4 for *E*. *japonica*; St. 11 for *E*. *strigata* in late April).

### 2.2 Sampling design to assess seasonal variances in the population densities and biomass of nymphs of two *Ephemera* mayfly species

**FIGURE 1:**
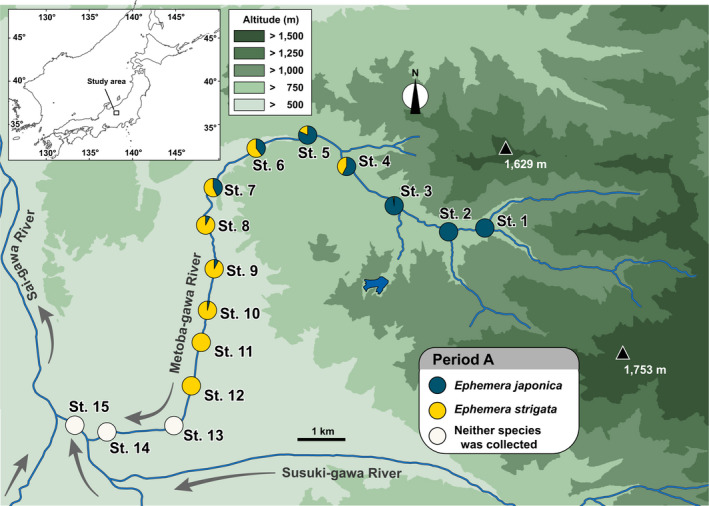
A map of Metoba‐gawa River, the target stream in this study, and the arrangement of the 15 fixed study sites. This map was made using QGIS ver. 3.10 (http://www.qgis.org/) and the digital elevation model (10 m digital elevation model, Source of reference is the Geospatial Information Authority of Japan; https://fgd.gsi.go.jp/download/menu.php). The pie charts on the map indicate the ratio of the two *Ephemera* mayfly species’ nymphs collected by quantitative sampling from each of the 15 research sites in late April (Period A). The resulting ratios observed of two species are displayed in Figure 3

**FIGURE 2:**
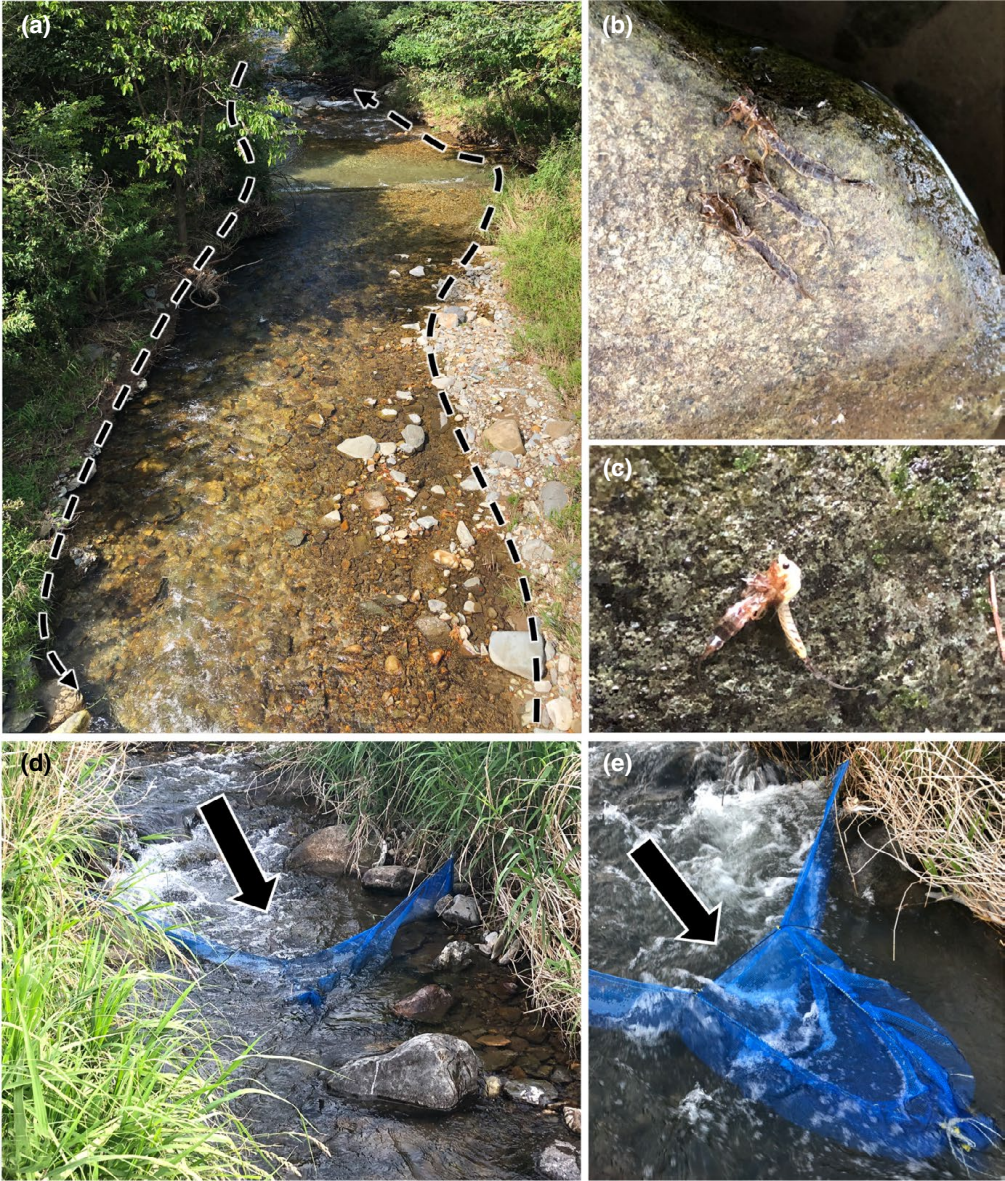
Methods for quantitative collection of emergence exuviae of *Ephemera japonica* and *Ephemera strigata*. (a) Transect lines (total 50 m) set up for quantitative collection for emergence exuviae of *E. japonica*. All observed exuviae were collected every day while walking along these lines. (b) Emergence exuviae of *E. japonica* observed on a boulder. (c) *Ephemera japonica* in the process of emergence on a boulder. (d) A runoff trap that crosses the flow path set up at St. 11 [3 m wide across the stream channel, while the length of the cross net along the channel was 0.8 m, the height (vertical height) of the net was 0.5 m and the mesh size was 5 mm] for the quantitative collection of the emergence exuviae of *E*. *strigata*. The top of this net was always set above the surface of the water flow. The black arrow exhibits flow direction. (e) An enlarged photo of the runoff net

We conducted a basic monthly quantitative survey from late April, before the emergence of *E*. *strigata*, to late September, after the emergence of *E*. *japonica* was completed, in 2019. This was done to evaluate the seasonal variations in the population densities and biomass of the two *Ephemera* mayflies in the Metoba‐gawa River. Unfortunately, due to the largest flood in the history of observation in October 2019, we discontinued our field research. At each of 15 research sites set up on the Metoba‐gawa River, five quadrats (0.30 × 0.30 m^2^) were set up in lotic water (riffle) environments and five quadrats were set up in slow current (lentic) environments including pools, after which quantitative sampling was carried out (i.e., the total number of quadrats was 150; Figures 1 and 2). Sampling of *Ephemera* mayflies using quadrats was performed by the same investigator each time, using the same standard net (mesh size: 1.0 mm). These quadrats were set up at least 1 m apart from each other. The location of each quadrat was accurately recorded by GPS located pegs driven into the riverbed, and marks (fluorescent tags) on river structures and trees on the stream bank. This preparation allowed for a carefully controlled quadrat survey throughout the entire April–September survey period. The collection numbers and wet weight (biomass) of *Ephemera* mayflies collected at each research site were calculated individually for each of the 10 quadrats.

To show that each of these quantitative surveys was carried out without problems, the daily precipitation, data from the Matsumoto Weather Observatory as published by the Japan Meteorological Agency was used.

In order to investigate the extent to which the environments inhabited by the two species of *Ephemera* mayflies overlap with each other in the Metoba‐gawa River, the elevation and riverbed gradient were evaluated using GIS at the 15 research sites. The riverbed slope degree was calculated using a GSI map and based on the distance between points at which there was a 10‐m elevation difference (basically, 5 m mesh map information from the “Geospatial Information Authority of Japan” was used for this analysis), with the study site set as the base point. In addition, substrate coarseness was evaluated in the same manner as our previous study (Okamoto et al., 2022). In evaluating the canopy openness rate, five digital hemispherical photographs were taken with a camera equipped with a fisheye lens (EX‐FR200, CASIO, Tokyo, Japan) at each research site, specifically at the position of the five quadrats in which the *Ephemera* mayflies were collected. Each photo was image‐analyzed using CanopOn2 software (http://takenaka‐akio.org/etc/canopon2/), and then averaged. In these image analyses, 10% of the image margin was removed in order to eliminate the influence of artificial objects such as skyscrapers as much as possible. DO (dissolved oxygen) and EC (electric conductivity), as water quality, were measured using DO meter (MM‐41DP, TOA DKK, Tokyo, Japan) and conductivity meter (Cyberscan CON 400, Nijkerk, Netherlands) respectively at each site. Also, pH, COD (Chemical Oxygen Demand), phosphate‐phosphorous (PO_4_^3−^), and the total Nitrogen concentration (NH_4_^+^–N or NH_3_^+^–N) were measured using four‐pack test kits (WAK‐pH, WAK‐COD, WAK‐TP and WAK‐TN, Kyoritsu Rika Kenkyu, Tokyo, Japan), after pumping out the stream water with a 100‐ml bottle.

Prior to conducting the quantitative research, the flow velocity and water depth at each quadrat sampling site were measured (5 replications per quadrat) in order to verify the environment on the microhabitat scale at the designated quadrat sites.

### 2.3 Sampling design to assess the respective emergence periods of the two *Ephemera* mayflies

In order to understand in detail the emergence periods of the two species, *E*. *strigata* and *E*. *japonica* in the Metoba‐gawa River, quantitative collection (i.e., a survey of the amount of exuviae of emerged mayflies collected flowing downstream within a certain of time, and counting the number of emerged exuviae observable while walking along a transect line of a certain length) of their molted emergence exuviae was carried out based on the emergence mode of each species (Figure 2). It is known that *E*. *japonica* emerges on land boulders/cobbles and *E*. *strigata* emerges on the surface of water (Ishiwata & Takemon, 2005; Okamoto et al., 2022; Takemon, 1989). In order to quantitatively collect the molted emergence exuviae of *E*. *japonica* that crawl up onto the gravel on the riverbank and emerge, two 25‐m transect lines (total 50 m) were set up at St. 4, and quantitative collection of the emergence exuviae attached to the gravel/cobbles at the water's edge was undertaken along these transect lines. Daily surveys of the numbers of emerged exuviae observed within the transect lines were conducted from June 7th to September 1st (Figure 2a–c). However, for the quantitative collection of the emergence exuviae of *E*. *strigata* that emerge on the water surface, a runoff trap that crossed the flow path was set up at St. 11 (so that the upper part of the net was always exposed from the water surface in the river) (Figure 2d,e). It was 3 m wide across the stream channel, while the length of the cross net along the channel was 0.8 m. The height (vertical height) of the net was 0.5 m and the mesh size was 5 mm. Sampling using this net was conducted from April 30th, before the emergence of *E*. *strigata* in the Metoba‐gawa River, to June 7th, after the emergence period was completed. The observed emergence exuviae were collected daily and the number of exuviae trapped were counted. From May 20th to May 21st, and on July 4th and 16th, emergence exuviae samples could not be collected due to flooding.

### 2.4 Data analysis

To examine the relationship between the distributions of *E*. *japonica* and *E*. *strigata* and environmental factors, Pearson's correlation coefficients were used to conduct density analyses of the two species with respect to each of the surveyed environmental factors (*cor* function in R package “psych”, Revelle, 2021; R software ver. 3.6.1, R Core Team, 2020).

Based on these datasets of late April (Period A), early June (Period B), and late June (Period C), whether density and biomass of the *E*. *japonica* differed, were tested statistically. The multiple comparison “Steel‐Dwass” test was performed to determine what combinations of seasons showed significant differentiation (“Steel‐Dwass test,” *pSDCFlig* function in R package “NSM3,” Schneider et al., 2020; R software ver. 3.6.1, R Core Team, 2020).

## 3 RESULTS

**FIGURE 3:**
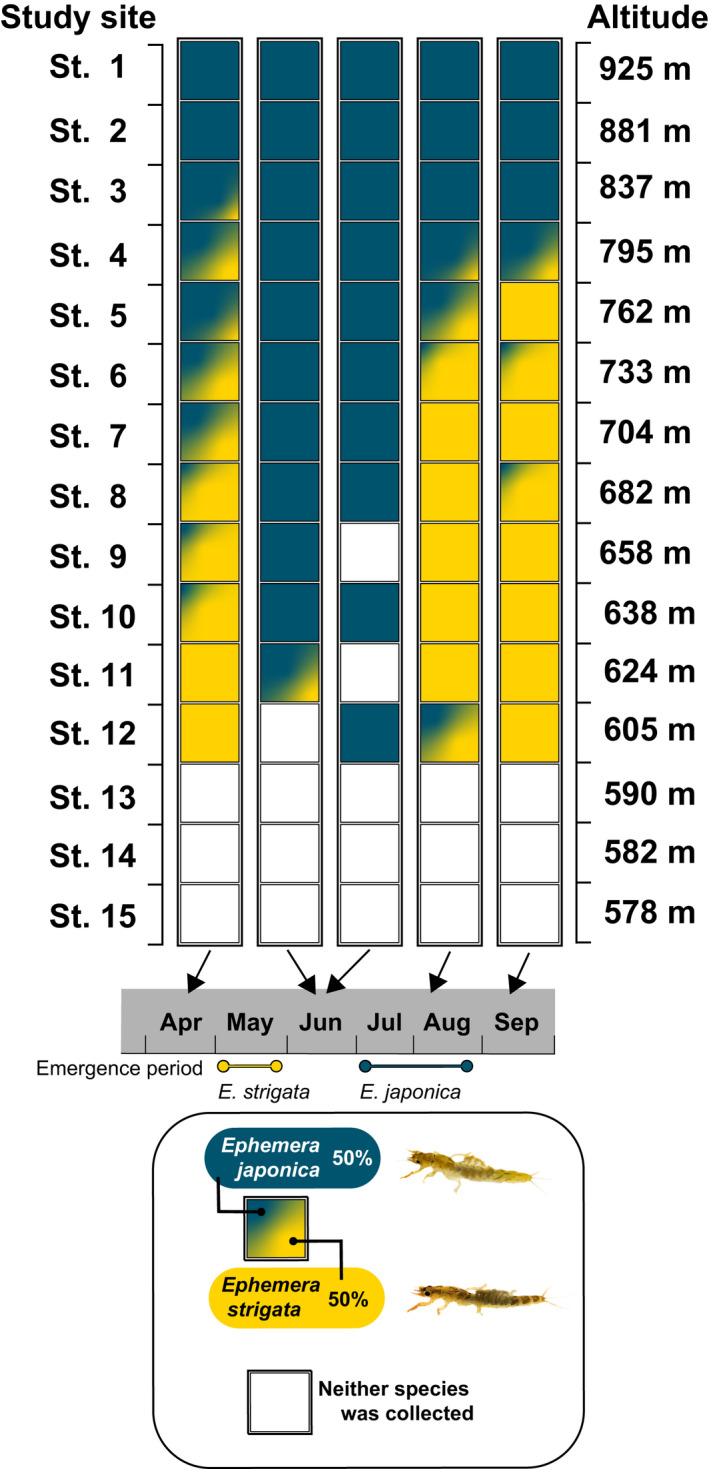
The proportions of collected *Ephemera japonica* and *Ephemera strigata* nymphs by our quantitative sampling, and their seasonal changes. The data shown in each square box (cell) indicate the total data of 10 quadrats at each study site, and the proportion of the two *Ephemera* species is relative to the collected numbers of specimens

**FIGURE 4:**
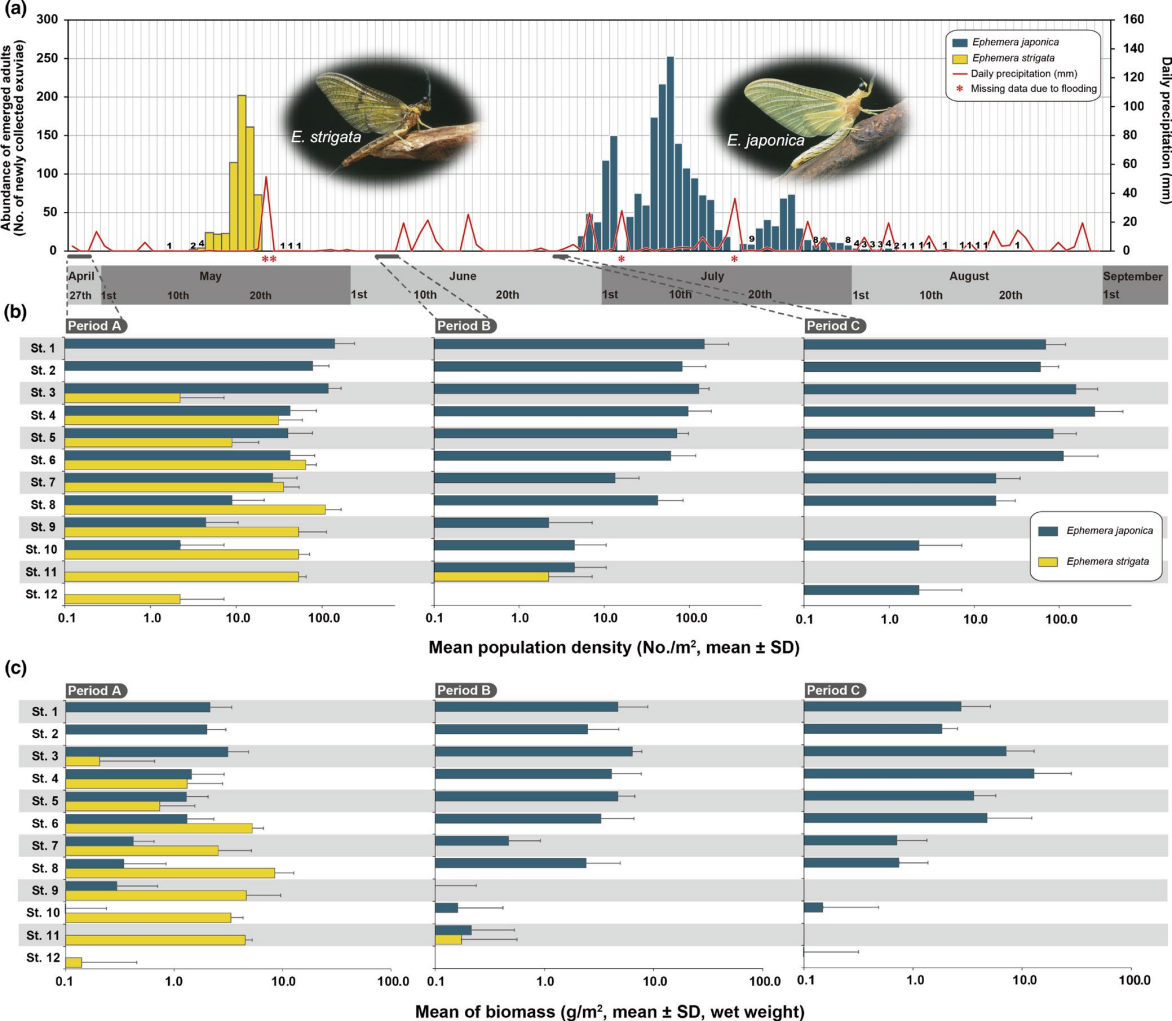
Life history and distribution patterns of *Ephemera japonica* and *Ephemera strigata* in the Metoba‐gawa River. (a) Emergence periods of *E. japonica* and *E*. *strigata*, and daily precipitation. If the number of exuviae collected was less than 10, since it is difficult to read the height of each bar, the number of specimens collected is shown. (b) Mean densities of two *Ephemera* species (nymphal stage) at each study site (No. of collected specimens/m^2^). (c) Mean biomass of two *Ephemera* species (nymphal stage) at each study sites (g/m^2^, wet weight). See Figure 5 for the fundamental data for each quadrat. For Figure 4b,c, the data of 5 quadrats set up at the lentic flows at each study site were used. This is because 90% of the specimens of the two *Ephemera* mayflies were collected from the lentic water environment through the Periods A, B, and C. For three study sites (i.e., St. 13–15), no graphs are shown in Figure 4b,c, as no *Ephemera* mayflies were collected within our quantitative sampling from any quadrats throughout all sampling seasons. The photographs of the just emerged subimago of two *Ephemera* mayflies were taken by Mr. Toshizo Karita

Results of quantitative sampling using quadrats for *Ephemera* nymphs in the Metoba‐gawa River are shown in Figures 3 and 4. In April (Period A), when surveying began, *Ephemera japonica* nymphs were collected at St. 1–10 and *Ephemera strigata* nymphs were collected at St. 3–12. That is, the habitats of these two species overlapped at St. 3–10. In the early and late June (Period B and C) surveys, most of the *E*. *strigata* nymphs were not collected, but were reconfirmed to be present in the August and September surveys. In the June period, when *E*. *strigata* nymphs were no longer collected, *E*. *japonica* nymphs were collected over a wide region (even at St. 11 and 12). Notably, *E*. *japonica* nymphs could be collected throughout the entire survey period, and they were collected in August even at St. 12. At St. 13–15, distribution of the *Ephemera* mayflies could not be confirmed (although in our preliminary survey and also our qualitative sampling of these sites, i.e., St. 13–15, only *E*. *strigata* was observed in small numbers). Table 1 shows the respective habitat characteristic ranges of the two *Ephemera* mayfly species collected in this study.

**TABLE 1:** Geographic information and measured environmental factors at each Metoba‐gawa River study site

Site no.	Latitude (N)	Longitude (E)	Altitude (m)	Degree of riverbed slope (%)	Substrate coarseness	Canopy openness (%)	DO (mg/L)	EC (mS/cm)	pH	COD (mg/L)	TP (mg/L)	TN (mg/L)	Mean of flow velocity (m/s, maximum–minimum)		Mean of depth (m, maximum–minimum)	
													*Ephemera japonica*	*Ephemera strigata*	*Ephemera japonica*	*Ephemera strigata*
St. 1	36.27293	138.03573	925	5.3	4.0	24.5	13.9	128.2	7.0	0	0.05	0	0.052 (000.2–0.157)	–	0.16 (0.06–0.30)	–
St. 2	36.27223	138.02904	881	6.4	3.8	41.7	13.9	161.3	7.5	0	0.05	0	0.054 (000.8–0.143)	–	0.19 (0.07–0.34)	–
St. 3	36.27704	138.01874	837	4.7	3.4	42.6	14.6	175.9	7.5	0	0.05	0	0.074 (000.2–0.281)	0.010	0.21 (0.06–0.42)	0.24
St. 4	36.28475	138.00954	795	3.3	4.0	37.6	14.3	176.5	7.5	0	0.05	0	0.057 (0.009–0.176)	0.091 (0.009–0.434)	0.26 (0.10–0.47)	0.27 (0.12–0.42)
St. 5	36.29057	138.00240	762	3.4	4.2	68.8	14.3	179.6	7.5	0	0.05	0	0.050 (0.002–0.328)	0.068 (0.002–0.153)	0.35 (0.15–0.54)	0.34 (0.12–0.52)
St. 6	36.28812	137.99237	733	2.7	4.0	51.9	14.3	197.6	7.5	0	0.05	0	0.035 (0.006–0.139)	0.099 (0.006–0.334)	0.19 (0.12–0.35)	0.28 (0.08–0.44)
St. 7	36.28054	137.98419	704	2.7	3.8	56.1	14.5	198.2	7.5	1	0.05	0	0.041 (0.008–0.188)	0.091 (0.008–0.224)	0.20 (0.03–0.35)	0.22 (0.14–0.31)
St. 8	36.27384	137.98286	682	3.0	3.3	85.8	14.7	199.4	7.5	0	0.05	0	0.054 (0.009–0.140)	0.134 (0.003–0.661)	0.33 (0.11–0.45)	0.28 (0.09–0.52)
St. 9	36.26509	137.98469	658	2.8	2.7	84.0	14.2	196.9	7.5	0	0.05	0	0.039 (0.005–0.072)	0.180 (0.005–0.682)	0.40 (0.31–0.48)	0.31 (0.11–0.58)
St. 10	36.25724	137.98314	638	2.3	3.8	82.3	14.7	232.0	7.5	0	0.05	0	0.048 (0.016–0.126)	0.099 (0.004–0.741)	0.18 (0.12–0.22)	0.25 (0.12–0.41)
St. 11	36.25153	137.98187	624	2.5	3.7	81.2	14.7	236.0	7.5	2	0.05	0	0.025 (0.005–0.060)	0.079 (0.008–0.225)	0.12 (0.06–0.14)	0.19 (0.11–0.32)
St. 12	36.24263	137.98014	605	1.9	2.6	82.2	14.9	203.2	7.5	2	0.05	2.5	0.023	0.056 (0.007–0.176)	0.28	0.29 (0.16–0.33)
St. 13	36.23524	137.97656	590	0.7	3.0	76.8*	13.6	228.0	7.5	0	0.05	0	–	–	–	–
St. 14	36.23419	137.96398	582	0.9	3.2	63.1*	13.3	250.0	7.5	0	0.05	2.5	–	–	–	–
St. 15	36.23582	137.95770	578	0.5	3.0	69.3*	13.7	194.6	7.5	0	0.05	2.5	–	–	–	

Note

*It seems that the rate of the canopy openness was underestimated because artificial objects (buildings, bridges, and others) in the city were reflected in the photograph. There were no sites with riparian forests.

**FIGURE 5:**
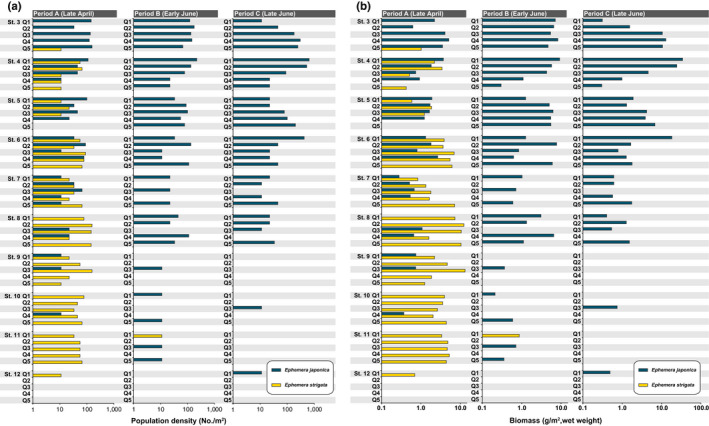
Seasonal density and biomass changes in the two *Ephemera* mayflies (*Ephemera japonica*, *Ephemera strigata*) for 5 fixed quadrats in St. 3–12, indicating a marked co‐habitation in the April survey. These are, the fundamental data for each quadrat are shown in Figure 4b,c. (a) density of two *Ephemera* species (nymphal stage), (b) biomass of two *Ephemera* species (nymphal stage)

The densities and biomasses of each species were examined at each site, and the densities and biomasses of *E*. *japonica* nymphs in Period A were found to be lower than their densities and biomasses in Periods B and C (Figure 4b,c). The results of the density and biomass survey data of each species of each quadrat in each research period at St. 3–12 are shown in Figure 5. When the densities and biomasses within the designated quadrat sites were analyzed, the densities and biomasses of *E*. *japonica* nymphs in Period B were generally higher than those of *E*. *japonica* in Period A (Figure 5a,b, Q1–2 in St. 4; Q1 in St. 6). In addition, the habitat densities and biomasses of *E*. *japonica* nymphs in Period C tended to be higher than those of *E*. *japonica* in Period A and B, respectively. Notably, this tendency was particularly remarkable at quadrats 1–2 (Q1–2) of St. 4 and Q1 of St. 6 (Figure 5a,b).

When we compared the results of environmental factor measurement in the cohabitation region of both *Ephemera* species in this study (Table 1) with the environmental factors of the “National Census on River Environments” administered by the Administrative Agency of the Ministry of Land, Infrastructure, Transport and Tourism of the Japanese government (Okamoto & Tojo, 2021), and also with the environmental factors of our previous study within the Asahi‐gawa River System (Okamoto et al., 2022), the result ranges in this study were almost completely within the ranges of the data trend in previous studies (Figure S1).

Between the four environmental factors (altitude, riverbed slop degree, canopy openness, and EC) and the densities of *E*. *japonica* nymphs in Periods A and B, a strong coefficient of correlation was observed (*r* ≥ .7). However, between densities of *E*. *strigata* nymphs and environmental factors the coefficients of correlation were not found to be so strong (Table S1).

To assess the emergence periods of the two *Ephemera* mayflies and identify their breeding seasons, emergence exuviae of *E*. *strigata* were collected from May 8th to May 24th (17 days), and the number of emergence exuviae collected on May 17th was the highest (Figure 4a). However, *E*. *japonica* exuviae were collected from June 8th to August 17th (ca. 70 days), and the number of emergence exuviae collected on July 9th was the highest (Figure 4a). The number of emerged *E*. *japonica* exuviae collected after July 31st did not exceed 10. Therefore, as there was a gap period of about one month between the breeding seasons of these two *Ephemera* mayflies, it became clear that they were significantly different from each other (Figure 4a).

**FIGURE 6:**
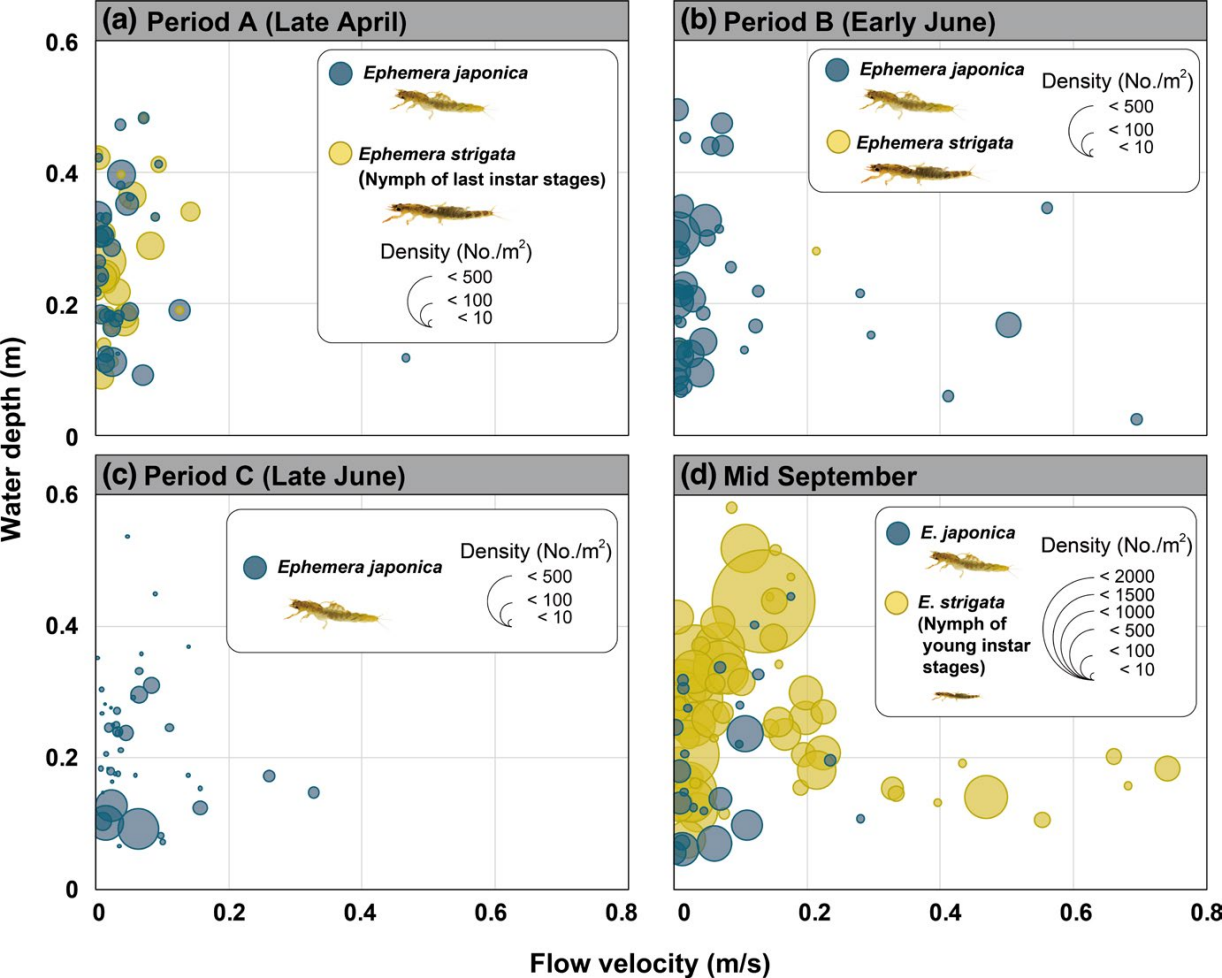
Relationships between water depth, flow velocity, and density [number of individuals collected at the level of each microhabitat (quadrat)] for the two *Ephemera* species. Data for 10 quadrats at each 15 study site are indicated as one plot, respectively

The result of the survey focusing on riffle and pool zones, even though both species were collected primarily in pools at each site during all three periods, a substantial number of *E*. *strigata* nymphs were also collected in the riffle zone only in August and September (Figure 6). In these periods, especially, a substantial number of *E*. *strigata* nymphs were found at St. 6–9 within the Metoba‐gawa River (Figure 6).

## 4 DISCUSSION

### 4.1 Fine‐scale longitudinal distribution pattern of two species of *Ephemera* mayflies in the Metoba‐gawa River

In the Metoba‐gawa River, although *Ephemera japonica* predominantly inhabits the upstream reaches compared to *Ephemera strigata*, a wide region of coexistence was also observed (Figures 1, 3 and 4b,c). Such a distribution pattern of these two *Ephemera* species is in good agreement with the trends reported in previous studies (Mizuno & Gose, 1993; Okamoto et al., 2022; Okamoto & Tojo, 2021; Takemon, 1989; Watanabe, 1985). In relationship between distribution of aquatic insect and environmental factors, generally, altitude and temperature are well known to be important factors in the distribution patterns of aquatic insects (Hildrew & Edington, 1979). In addition, as for *Ephemera* mayflies, it has been reported that the riverbed gradient is also closely related to their distribution patterns (Okamoto & Tojo, 2021; Watanabe, 1985). In addition, the relationship between the distribution of *E*. *japonica* nymphs in the Asahi‐gawa River System and canopy openness has previously been supported by means of a redundancy analysis (Okamoto et al., 2022).

The results of in this study on canopy openness in the habitats of *E*. *japonica* nymphs were generally small percentages (i.e., riparian forests are comparatively well developed), similar to the trends of the Asahi‐gawa River System research in our previous study (Okamoto et al., 2022). However, in this survey within the Metoba‐gawa River, *E*. *japonica* nymphs could be collected even in relatively open areas (at several sites that were not covered by the canopy; i.e., about 50%–86% canopy openness at St. 5–8). This is because the Metoba‐gawa River is a tributary in the uppermost regions of the Chikuma‐Shinano‐gawa River System, but a relatively large city (Matsumoto City) has developed in this basin, and as a result the riparian forest has been cut down. Even at these study sites (i.e., comparatively high canopy openness sites), the characteristics of the upstream areas were recognized according to the measured values of other environmental factors (Table 1).

The distributional area of aquatic insects is often strongly associated with water quality (Jiang et al., 2011; Sabha et al., 2020). However, the results of water quality analyses in this study did not show any significant relationship in the determination of the distribution the two species (DO, EC, pH, COD, TP, TN). This is because, this study has been conducted on a geographically fine scale, and because there are no in flowing tributaries from alternate water sources within the survey area. The correlation between EC values and the density of *E*. *japonica* nymphs in both late April (Period A) and early June (Period B) were strong. However, no difference was observed in that tolerance to water quality between the two species in their overlapping areas of habitation in the up‐stream to middle‐stream region. This is because the distributional area of *E*. *japonica* nymphs overlapped with distributional area of *E*. *strigata* nymphs, throughout the entire survey period.

In conclusion, it was related that the niche of both species is widely overlapping, however, a tendency for each of the two species of the *Ephemera* mayflies to taper gradually inversely proportionate to the other's density within this geographically fine scale is scarcely detectable. With this as a premise, we will discuss seasonal fluctuations in the distribution areas and interspecific interactions in the following section.

### 4.2 Spatiotemporal variation in the longitudinal distribution patterns of these two *Ephemera* mayflies

Although it is well known that the habitats of aquatic insects are often temporarily displaced due to disturbances such as flooding (Poff et al., 2018), the degree of flooding that occurred during this study was not enough to displace these mayfly habitats (Figure 4a). However, the impact and disturbance of the large‐scale flood that occurred immediately after this (mid October 2019) was enormous, and the environments at the 15 survey sites’ set up changed significantly, so unfortunately it was not possible to continue tracking seasonal fluctuations after the flood.

In Period A (27th to 30th April), which preceded the emergence seasons of both *Ephemera* species, the distribution areas of nymphs of both *Ephemera* mayflies overlapped widely (Figures 3 and 4b,c). These results are consistent with a tendency reported by Okamoto et al. (2022). However, when we analyzed in detail the species composition and biomass of each species for each of the 10 quadrats set up at each research site, it was confirmed that there were quadrats that recorded both species (i.e., Period A, Q1–4 in St. 4; Q1–3 in St. 5; Figure 5), and also quadrats in which only one species was collected (i.e., Period A, Q5 in St. 4; Q4 in St. 5; Q5 in St. 6; Figure 5). These results show that both *Ephemera* species can utilize the same space on a very fine scale (i.e., microhabitat).

In Period B (3rd to 4th June) and Period C (26th to 27th June), few *E*. *strigata* nymphs were collected. This period was immediately after the breeding season of *E*. *strigata*, and since it corresponds to the egg and young instar nymphal stage(s) within the lifecycle of *E*. *strigata*, it was thus not possible to conduct quantitative collections in this study (Figures 4 and 5). It was also revealed that *E*. *japonica* nymphs immediately filled the resulting unoccupied spaces (open niches) in Periods B and C, from which the large *E*. *strigata* nymphs had disappeared due to their having emerged (Figures 3, 4, 5). Although densities and biomass of *E*. *japonica* in quantitative sampling among these three study periods (i.e., Period A, B and C) were tested by multiple comparisons, none exhibit statistically significant differences (Steel‐Dwass test, *p* > .05). However, it is considered to be an important result that before the emergence of *E*. *strigata* (i.e., Period A), *E*. *japonica* nymphs could not be collected from St. 11 and St. 12, whereas after the emergence of *E*. *strigata* (i.e., Periods B and C), *E*. *japonica* nymphs could be collected from both sites (i.e., St. 11 and St. 12; Figure 3). Thus, it seems clear that *E*. *japonica* nymphs were newly distributed and expanding within the newly unoccupied spaces (i.e., newly vacant niches) following the emergence of *E*. *strigata* (Figure 4b,c).

The morphology, behavior, and physiological traits of closely related species are often similar, and the available habitat and resources available are also often similar. In order to enable the coexistence of species, niche differentiation occurs, and as a result, habitat segregation is often observed. Yet, the targeted ephemerid species have been considered to have adapted to different environments in different river systems (Okamoto et al., 2022; Okamoto & Tojo, 2021). In this study, the habitat type used by one ephemerid species was at times found to be inhabited by the other species on a local scale. Hence, the adapted environments of each species are not segregated completely, that is, they are partly overlapped. In the case of these two closely related species of mayfly (i.e., *E*. *japonica* and *E*. *strigata*) which we focus on in this study, “seasonal segregation” may have caused their niche differentiation. In fact, when one species *E*. *strigata* emerges it creates a niche gap, of which the other species *E*. *japonica* quickly takes advantage. The background to this possibility is the movement of habitats due to “flow‐down,” when the environment provides a lack of suitable habitats. The behavior of utilizing the flow‐down strategy to find a suitable habitat may have contributed to their seasonal habitat segregation and seasonal changes in their distribution ranges. And such a dispersal also constitutes a “temporary distribution area expansion” and may be an important feature contributing to niche differentiation. Therefore, it is highly probable that this phenomenon revealed in this study constitutes an important mechanism by which the coexistence of multiple species is able to occur within a limited geographical space.

In dragonflies (Suhling, 1996), it is known that they adapt to make use of another habitat, if other species (including other genus) that prefer their initial habitat and come to populate it in higher density. In such circumstances, the occurrence of interspecific interactions have been considered. In this study, however, such habitat selectivity changes between the two target species in the habitats classified as the riffle zone and pool zone could not be detected in the situations where the two species were in coexistence. Since ephemerid mayflies have a “burrowing” lifestyle, substrate characteristics should strongly contribute to their habitat preferences (Hwang et al., 2013; Ishiwata & Takemon, 2005; Okamoto et al., 2022; Sun & Chang, 2016). Therefore, it may be possible to evaluate the preferences for gravel size as shown by the experimental study by Suhling (1996) on burrowing dragonflies of the genera *Onychogomphus* and *Gomphus*. In the future, it may be possible to more clearly show the interspecific interactions in the target species by means of experimental manipulation (e.g., remove experiments, Connell, 1961a, 1961b; Wise, 1981; Paterson et al., 2018).

During the breeding season of *E*. *strigata*, it is known that mated *E*. *strigata* females fly upstream for oviposition, and they prefer to lay eggs in microhabitats on the upstream side of riffle zones (Tanaka et al., 2003). However, *E*. *japonica* oviposits little by little in multiple pools while flying upstream (Takenaka, unpublished observation in the same Metoba‐gawa River; Tojo has also observed similar phenomena in various mountain streams). In this study, it was also revealed that only small nymphs of *E*. *strigata* inhabit the relatively high‐velocity microhabitats (Figure 6). This tendency is thought to be related to the oviposition behavior of *E*. *strigata* as mentioned above. The fact that the distribution ranges of large *E*. *strigata* nymphs shifted slightly downstream before their breeding season is thought to be influenced by their interspecific interaction with *E*. *japonica*.

Traditionally, these two *Ephemera* species were understood to have only slightly different habitat preferences, and it had been thought that such a minor niche differentiation was the sole basis of their habitat segregation (Okamoto et al., 2022; Okamoto & Tojo, 2021). However, the findings of this study demonstrate that interspecific interactions between these two species also actively contribute to their habitat segregation patterns. Although data were collected over time, our previous study results on the longitudinal distribution patterns of *Ephemera* mayflies in another river system (i.e., the Asahi‐gawa River System) suggested that the population densities of both species, *E*. *japonica* and *E*. *strigata*, have an inverse relationship with each other (Okamoto et al., 2022). The results of this study at the Metoba‐gawa River are consistent with the results of our previous study.

In addition, although there are only a few cases, such fine‐scale (microhabitat scale) habitat conflicts among aquatic insects of the same feeding function group have been reported: interspatial competition for microhabitats between a *Hydropsyche* caddisfly and a black fly (Hemphill, 1988), and competition for nesting sites between two *Stenopsyche* caddisfly species, *Stenopscyche marmorata* and *Stenopsyche sauteri* (Funakoshi, 2005). This study's observation of *Ephemera* mayflies in the Metoba‐gawa River is considered to be a significantly valuable finding regarding interspecific interactions between closely related and sister species of the same feeding function group.

This study of *Ephemera* mayflies suggests that in the overlapping distribution region between closely related species, the seasonal occupancy of one species may limit the distribution of the other species. It has been suggested that interactions between organisms on a local geographic scale may also affect their respective distribution on a larger geographic scale (Case et al., 2005; Wisz et al., 2013). In distribution pattern formation in aquatic insects with large restrictions on migration and dispersion (Takenaka & Tojo, 2019; Takenaka et al., 2019; Tojo et al., 2017), the role of interspecific interactions on the microhabitat scale between such closely related species may be greater than previously estimated. This result will contribute to the accumulation of new findings on the formation of species distributions in freshwater ecosystems.

## AUTHOR CONTRIBUTIONS

**Seiya Okamoto:** Conceptualization (equal); Data curation (equal); Formal analysis (lead); Investigation (lead); Methodology (equal); Visualization (lead); Writing – original draft (equal); Writing – review & editing (equal). **Masaki Takenaka:** Conceptualization (equal); Data curation (equal); Investigation (supporting); Methodology (equal); Visualization (equal); Writing – review & editing (equal). **Koji Tojo:** Conceptualization (lead); Data curation (equal); Funding acquisition (lead); Methodology (lead); Project administration (lead); Resources (lead); Supervision (lead); Visualization (lead); Writing – original draft (lead); Writing – review & editing (lead).

## Supporting information

Supplementary Material

## Data Availability

The authors confirm that the data supporting the findings of this study are available within the article and in its supplementary data.
