# Supplementary material for: Seasonal modifications of longitudinal distribution patterns within a stream: Interspecific interactions in the niche overlap zones of two Ephemera mayflies
Source: Ecol Evol. 2022 Apr 1;12(4):e8766. doi: 10.1002/ece3.8766 (PMC8975782; doi:10.1002/ece3.8766)
Supplement: Supplementary file 1 — Supplementary Material [file ECE3-12-e8766-s001.docx]

**Supplemental information**

***Ecology and Evolution***

**Seasonal modifications of longitudinal distribution patterns within a stream: Interspecific interactions in the niche overlap zones of two *Ephemera* mayflies**

**Seiya Okamoto^1^・Masaki Takenaka^2,3^・Koji Tojo^1,3,4*^**

^1^ Division of Mountain and Environmental Science, Interdisciplinary Graduate School of Science and Technology, Shinshu University, Asahi 3-1-1, Matsumoto, Nagano 390-8621, Japan

^2^ Sugadaira Research Station, Mountain Science Center, University of Tsukuba, Sugadairakougen 1278-294, Ueda, Nagano, Japan

^3^ Department of Biology, Faculty of Science, Shinshu University, Asahi 3-1-1, Matsumoto, Nagano 390-8621, Japan

^4^ Institute of Mountain Science, Shinshu University, Asahi 3-1-1, Matsumoto, Nagano 390-8621, Japan

*Corresponding Author: Koji TOJO, e-mail: ktojo@shinshu-u.ac.jp

**TABLE S1** Pearson’s correlation coefficients were calculated combining all environmental factors as recorded at each study site, and the mean density of *Ephemera japonica* and *Ephemera strigata* in each season. The gray cells show a *r* ≥ 0.7 coefficient of correlation

**
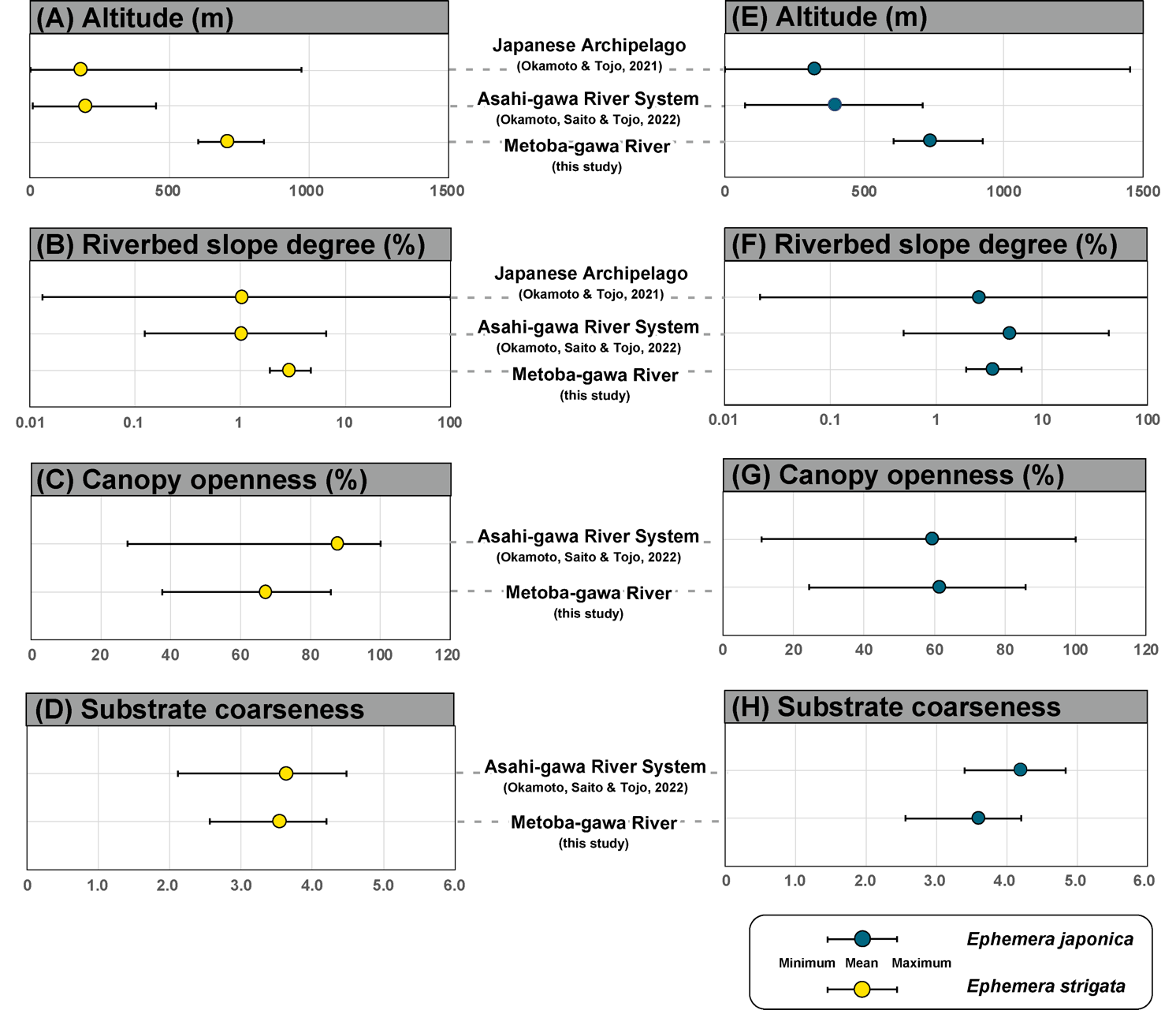
**

**FIGURE S1** Comparison of habitat characteristics (altitude, riverbed slope degree, canopy openness and substrate coarseness) of *Ephemera* species in this study with those in previous studies. A–D: environmental factors for habitat characteristics of *Ephemera strigata*, E–H: environmental factors for habitat characteristics of *Ephemera japonica*
